# A Cross-Sectional Pilot Study to Examine the Criterion Validity of the Modified Shuttle Test-Paeds as a Measure of Cardiorespiratory Fitness in Children

**DOI:** 10.3390/ijerph15102290

**Published:** 2018-10-18

**Authors:** Nikki Milne, Michael J. Simmonds, Wayne Hing

**Affiliations:** 1 Faculty of Health Sciences and Medicine, Bond University, Robina, QLD 4226, Australia; whing@bond.edu.au; 2 Menzies Health Institute Queensland, Griffith University, Gold Coast, QLD 4220, Australia; m.simmonds@griffith.edu.au

**Keywords:** child, obesity, cardiorespiratory, fitness, health, assessment

## Abstract

With accumulating evidence that exercise capacity decreases all-cause mortality independent of adiposity, benefits may be gained by developing cardiorespiratory fitness measures that are specifically and sensitively designed for use with pediatric populations when cardiorespiratory fitness may be a contributing factor for obesity. This study aimed to examine the criterion validity of the Modified Shuttle Test-Paeds (MSTP) as a measure of cardiorespiratory fitness in children, against the gold-standard reference; VO_2_peak, compared to the commonly used field-test; 20-m Multi-Stage-Shuttle-Run-Test (20-m MSRT). A cross-sectional pilot study, with 25 school-aged children (age: 6–16 year; male/female: 19/5; BMI: 21 ± 9 kg/m^2^) was employed. Physical measures included: Bruininks-Oseretsky-Test-of-Motor-Proficiency-2nd Edition (BOT2), VO_2_peak, 20-m MSRT, MSTP, body composition/anthropometry. The mean cardiorespiratory fitness of participants was: VO_2_peak: 43.8 ± 11.2 (mL/kg/min); 20-m MSRT: 5.48 ± 2.96 (level); MSTP: 22.10 ± 3.05 (no.). A strong predictive relationship was found between the 20-m MSRT and VO_2_peak (r^2^ = 0.486, *p* < 0.001) whereas a very strong predictive relationship existed between the newly designed MSTP and VO_2_peak (r^2^ = 0.749, *p* < 0.001). Whilst further research with larger study cohorts is needed, this pilot study found the MSTP to have a very high predictive validity for estimating VO_2_peak in children, suggesting it may be a valid child-specific indicator of cardiorespiratory fitness requiring only a simple equation that is clinically relevant.

## 1. Introduction

For many years, population-wide surveys have consistently indicated increased adiposity as well as decreased cardiorespiratory fitness in pediatric populations, with approximately 0.5% and 1% per year reductions in performance on field-based cardiorespiratory fitness tests for children and adolescents respectively [1]. These longitudinal trends indicate declining functional capacity for children during weight-bearing activities [2]. Difficulty performing weight-bearing activities will likely limit involvement in recreational physical activity such as lunch-time play, and/or organized sport, further compounding the decreased cardiorespiratory fitness of these children. This spiraling “chicken and egg” effect could consequently impact learning and cognitive performance [3,4] and lead to chronic disease in adulthood [5]. With accumulating evidence that exercise capacity decreases all-cause mortality [6] independent of adiposity [7], there are clear benefits to be gained through the development of cardiorespiratory fitness measures that are specifically and sensitively designed for use with pediatric populations. Such tools could assist with detecting children who would benefit from early intervention to improve cardiorespiratory fitness, decrease the likelihood of developing obesity and prevent subsequent health and educational impairments. 

Peak oxygen uptake (VO_2_peak), the highest VO_2_ elicited during a given exercise bout to exhaustion, is widely accepted as a valid marker of cardiorespiratory fitness in children [8]. However, laboratory testing to determine VO_2_peak is not commonly used with children in health professional clinics or field environments as it requires expensive equipment and specialist skills to collect and analyze the data. A measure of cardiorespiratory fitness which is more frequently used in school or community environments is the 20-m Multistage-shuttle-run-test (20-m MSRT) [9,10]. This measure can take up to 20 min to complete, depending on fitness level and its popularity can be attributed to its practical use for simultaneous measurement of large groups. However, when used in large groups, the level of achievement becomes obvious to peers, as each individual “drops out” at the point of exhaustion, exposing children who are less fit to potential for stigmatization by peers. As such, a cardiorespiratory fitness measure which is inexpensive, shorter in duration, does not require specialist clinical skills, does not have a “drop out” nature and requires less physical space, making it more suited to screening the fitness of children in schools or health professional environments is warranted. 

The Modified-Shuttle-Test-Paeds (MSTP) has been specifically and sensitively designed to address the limitations of current cardiorespiratory testing methods. It involves a task-oriented approach while the child runs a 10-m shuttle, picks up a hand-held bean bag, turns around and returns to the start point to place the hand-held bean bag in a tray. This is repeated as many times as possible in three minutes. The test can be performed individually or in class groups, whereby all children are instructed to perform at maximal effort for the full three minutes with strong verbal encouragement offered by the examiner. This study therefore aims to: (1) test the concurrent and predictive validity of the MSTP as a measure of cardiorespiratory fitness in children, against the gold standard reference: VO_2_peak and; (2) Contrast the strength of the relationship between the MSTP and VO_2_peak, with that of the 20-m MSRT. 

## 2. Materials and Methods

### 2.1. Participants

Twenty-five children (age: 6–16 year; male/female: 19/5; BMI: 21 ± 9 kg/m^2^) consented to participate in the present study after advertisement to local schools and through community flyers. Eligible participants were aged 5 to 17 years inclusive and attended school. Children diagnosed with mobility-limiting orthopedic conditions, cardiac, non-reversible pulmonary or neurological conditions were excluded from participation. Informed consent and a parent completed medical history form were obtained by the parents of the children wishing to participate in the study and all children provided assent to indicate their willingness to participate. 

### 2.2. Experimental Design

In this cross-sectional observational study, participants were refreshed with the experimental procedures and familiarized with the equipment prior to their test day which for all children occurred in the summer of 2013. Basic anthropometry, clinical evaluations, motor proficiency, and various indices of cardiorespiratory fitness were measured. All children were encouraged to have a light breakfast 2 h before their appointment. Heart rate and blood pressure were measured in a seated position after 10 min of quiet rest upon arriving at the laboratory. Height, weight and bioimpedance measures were subsequently measured, before each participant completed the first cardiorespiratory fitness test; the 20-m MSRT. Following the completion of the 20-m MSRT, a one-hour washout period was implemented to allow the participant to recover to a resting state. Apart from the 20-m MSRT always being scheduled as the first of the cardiorespiratory fitness measures (due to room booking restrictions), all other measurements were randomly assigned to each participant with special consideration of implementing washout periods of no less than 1 h between strenuous assessments. Five participants required a physician to be present during incremental exercise testing due to being considered ‘higher risk’ for exercise-induced complications, which included those who were obese (BMI > 95th percentile; *n* = 3) or presented with asthma (*n* = 2). Ten minutes after completing each of the MSTP, 20-m MSRT, incremental exercise test, and the BOT2, participants rated their “likeability” of each assessment using a simple visual feedback tool, using “emoticon” faces linked to a written scale of: 1. Fantastic; 2. Great; 3. Okay; 4. Boring; 5. Upset. This visual likert scale measure was created for this study to quickly monitor the affective valence or emotional stress that the tests may potentially have caused the children. A registered health professional, trained to work specifically with children, was available to debrief with the participants should they indicate the rating of “upset” for any assessment and to ensure that they were happy to continue their participation in the study. Bond University Human Research Ethics Committee reviewed and approved the experimental protocol for the present study (RO1601). All research was conducted in accordance with the Declaration of Helsinki (1964). Age, anthropometric measures, body composition and motor skill proficiency were all noted to be possible confounding factors in the relationships to be explored in this study and for this reason they were measured with all participants.

### 2.3. Anthropometric and Body Composition Measures

Standing height was measured barefooted at the top of a tidal inspiration on a solid surface using a tape measure to the nearest 0.5 cm. Body mass was measured while participants wore light clothing (e.g., shirt and shorts) using a body composition analyzer (MC-980MA, Tanita Corporation, Tokyo, Japan). Participants were blinded to their body composition through the implementation of a barrier-screen over the instruments digital display. 

### 2.4. Motor Skill Proficiency

The BOT2 [11] is a valid and reliable diagnostic and evaluative tool for children and young adults aged 4.5–21 years, that was used to characterize motor proficiency of participants to ascertain if the study cohort was representative of the Australian population of children. It also acted as a pretest for any children who continued into the intervention, a second stage of this study (not reported in the present paper). Within the BOT2, Fine Motor Precision and Fine Motor Integration subtests were combined to make up Fine Manual Control. Manual Dexterity and Upper Limb Coordination subtests were combined to make up Manual Coordination. Bilateral Coordination and Balance subtests were combined to make up Body Coordination. Running Speed and Agility and Strength were combined to make up Strength and Agility. Fine Manual Control, Manual Coordination, Body Coordination and Strength and Agility composite scores were summed to make up Total Motor Proficiency. The study protocol required the BOT2 to be broken up into fine and gross motor stations for testing. Exceptions to the standardized instructions included the order of testing, which was scheduled around the cardiorespiratory fitness measures and washout periods. 

### 2.5. Cardiorespiratory Fitness Measures

#### 2.5.1. 20-m Multi-Stage Shuttle Run Test (20-m MSRT) Protocol

The 20-m MSRT was completed by all participants. This valid and reliable field test [10] is a commonly utilized assessment of cardiorespiratory fitness. The protocol for this measure included the participants being instructed to repeatedly run between two markers placed 20 m apart until volitional fatigue. The 20-m MSRT progressively increases intensity, with the pace of the test indicated by audible tones on a music player, commencing at a speed of 8.0 km/h which is increased by 0.5 km/h each stage after the first minute. This test was conducted in an indoor environment, and strong verbal encouragement was provided throughout the test to increase the likelihood of a maximal effort. The test was terminated when a participant failed to reach the 20 m marker on two consecutive shuttle runs (laps). The outcome measure was the total number of laps completed for each individual, which was subsequently converted to a standardized score (level completed) and then converted to predicted VO_2_peak using the predictive equation previously published by Leger et al. [10]. 

#### 2.5.2. Modified Shuttle Test-Paeds (MSTP) Protocol

The MSTP was completed by all participants. Participants were instructed to run a straight 10-m shuttle, pick up 1 hand-held bean bag from a tray (10 cm high × 40 cm width, 30 cm length) on the ground, turn around and return to the start point to place the hand-held bean bag into an identical size tray and repeat this task as many times as possible in three minutes. The test was conducted in an indoor environment on the same surface as the 20-m MSRT. Children were encouraged strongly throughout the test with verbal encouragement, particularly towards the end to ensure maximal effort was achieved. One (1) point was scored for every bean bag returned to the tray at the three-minute completion time. A further half point was added to the score if the test finished after the child had picked up a bean bag, but they had not yet returned it to the tray. 

#### 2.5.3. Determination of Peak Oxygen Uptake and Ventilatory Thresholds

Participants performed an incremental exercise test to volitional fatigue on a motor driven treadmill (‘Valiant’; Lode B.V., Groningen, The Netherlands) for the determination of peak oxygen uptake (VO_2_peak), time to exhaustion and ventilatory thresholds. Participants commenced exercise at a predetermined preferred walking speed of 4.0–5.0 km/h at 0% grade for 4 min, before the speed was increased every 60 s until the participant achieved their previously determined preferred running speed (6.0–8.0 km/h). To avoid prolonged testing and to facilitate expected test duration < 12 min treadmill grade was then increased by 1% (younger participants) or 2% (adolescents) every 60 s [12], with strong verbal encouragement at each level, until volitional fatigue or signs and symptoms precluded further exercise. Brachial artery blood pressure was measured and recorded every three minutes and cardiac rhythm was monitored using a 12-lead ECG (Cardio Perfect, Welch Allyn Inc., Skaneateles Falls, NY, USA) during the incremental exercise test. Oxygen uptake, carbon dioxide output (VCO_2_), and minute expired ventilation were measured breath-by-breath using a calibrated open-circuit metabolic measurement system (Ultima CPX, Medical Graphics Corporation, St Paul, MN, USA). Peak exercise values were determined as the average of the two highest consecutive 30 s values measured before volitional fatigue. The ventilatory thresholds were determined according to methods previously described in the literature [13,14]. 

### 2.6. Statistical Analysis

Means, standard deviations and bivariate correlations were calculated for all physiological, anthropometrical and aerobic fitness measures. After assumptions of normality and linearity were tested, independent samples t tests were used to assess cross-sectional differences in measured variables between groups for gender. Pearson’s product moment correlations were performed to assess the relationship between participants’ physiological and anthropometrical characteristics and measures of cardiorespiratory fitness. Simple linear regression analysis was used to determine the degree by which the MSTP Total score could predict cardiorespiratory fitness; VO_2_peak (mL/kg/min) and Ventilatory Thresholds 1 (VT1) and 2 (VT2). Multiple regression analysis was then used to determine if the predictive validity of the MSTP for estimating VO_2_peak could be further strengthened by the addition of other possible confounding variables (e.g., age, gender, weight and height) and to establish a prediction equation of VO_2_peak from the MSTP. Statistics were analyzed with SPSS for Windows (Version 23.0, IBM Corp., Armonk, NY, USA). Significance level was set at *p* < 0.05. Power analyses were performed using an effect size calculator (Polytechnic University, Hung Hom, Kowloon, Hong Kong) [15] and G-power Software (Version 3.1.7, Heinrich Heine University, Dusseldorf, Germany). This was performed post-hoc as no data previously existed for the MSTP. A power analysis for correlation statistics revealed that with a sample of 24 participants, and a 2-talied α value of 0.05 and an expected effect size of at least r = 0.6, a statistical power of 94% can be achieved. Finally, to test the null hypothesis that distributions of responses for the Test of Likeability were not significantly different across the measures of BOT2, VO_2_peak, MSTP and 20-m MSRT, the Kruskal-Wallis test was applied. 

## 3. Results

### 3.1. Participant Characteristics

Twenty-five children ranging from underweight (*n* = 3) to morbidly obese (*n* = 3) participated in this study. One child did not complete VO_2_peak testing on the treadmill due to the identification of a cardiac arrhythmia that precluded further exercise testing. Six (24%) children in this study were classified as overweight or obese using the Centers for Disease Control and Prevention (CDC) BMI percentile ranges (≥85th percentile) [16]. This figure is consistent with the Australian population of approximately 1 in 4 children being overweight or obese [17]. The mean BMI percentile for boys was 51.58 ± 36.27 and for girls was 52.67 ± 28.19, placing the means for both genders in the ‘healthy weight’ range. The mean Total Motor Proficiency Percentile Rank for children in the study was 55.24 ± 33.13, indicating that overall the children in this study had ‘average’ motor skill ability with a wide range of motor proficiency from ‘low’ to ‘very high’. 

Table 1 outlines the physiological and anthropometric characteristics of study participants and correlations of these measures to cardiorespiratory fitness results. For the present study, the MSTP, 20-m MSRT and VO_2_peak expressed in mL/kg/min, had no significant relationships with age or height. Body mass but more so BMI (raw scores) were shown to have moderate to high significant negative correlations with VO_2_ peak (mL/kg/min) and MSTP but these relationships were not significant with the 20-m MSRT. 

The cardiorespiratory fitness characteristics of the study participants are summarized in Table 2. Statistical analysis using t-tests for investigating equality of means demonstrated no significant differences between boys and girls in mean MSTP scores (M: 22.21 ± 3.41, F: 21.75 ± 1.60, t = −0.316, DF = 23, *p* = 0.755), 20-m MSRT (M: 6.11 ± 3.07, F: 3.5 ± 1.38, t = −1.993, DF = 23, *p* = 0.058) or the directly measured VO_2_peak (M: 44.65 ± 12.78, F: 41.32 ± 3.55, t = −0.622, DF = 22, *p* = 0.540).

### 3.2. Concurrent and Predictive Validity of the MSTP as a Cardiorespiratory Fitness Measure 

To establish the concurrent validity of the MSTP and to determine the strength of the relationship of the MSTP to VO_2_peak as well as the 20-m MSRT to VO_2_peak, Pearson’s correlations were undertaken (see Table 3). The results demonstrated that the MSTP total score was significantly and very strongly correlated with the gold standard measure of cardiorespiratory fitness: VO_2_peak. 

To determine the predictive validity of the MSTP for approximating VO_2_peak (mL/kg/min), regression analyses were also undertaken (see Table 3). The MSTP was found to have a very high predictive validity for estimating VO_2_peak, accounting for 75% of the variance in VO_2_peak for children in this study, with a standard error of the estimate of 5.64 mL/kg/min or 12.78%. The 20-m MSRT accounted for 49% of the variance in VO_2_peak, with a standard error of the estimate of 8.08 mL/kg/min or 18.31%. To determine if the addition of other possible confounding variables could further strengthen the predictive validity of the MSTP in this study, a multiple regression analysis was undertaken and showed that in the presence of MSTP; gender, age, weight and height were not significant predictors of VO_2_peak. VO_2_peak alone could be validly predicted using the MSTP scores of children in this study by applying the following equation:

[VO_2_peak (mL/kg/min) = (−24.5 + (3.122 × MSTP Total Score))] (1)

**Figure 1:**
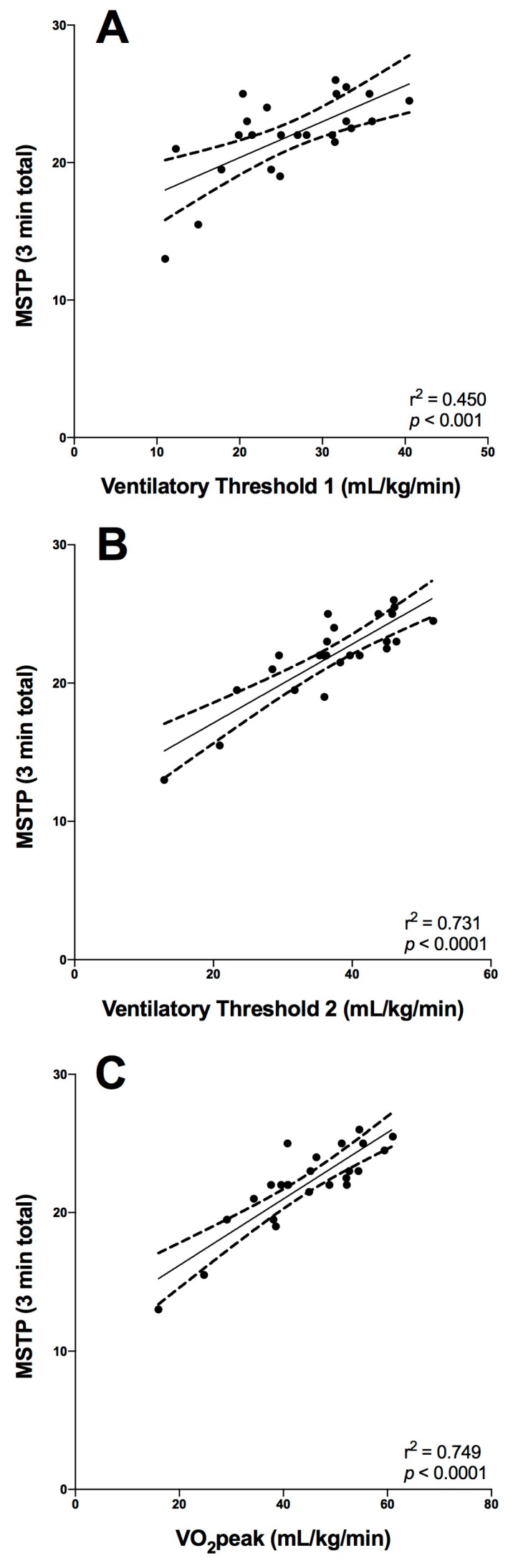
Relationship of the Modified Shuttle Test-Paeds (MSTP) to important parameters of cardiorespiratory fitness; (**A**) Ventilatory Threshold 1 (VT1), (**B**) Ventilatory Threshold 2 (VT2) and, (**C**) Peak Oxygen Uptake (VO_2_peak).

As Ventilatory threshold 1 (VT1) and 2 (VT2), along with VO_2_peak are all important parameters of cardiorespiratory fitness, a regression analysis was performed investigating their relationship with the MSTP. Figure 1 outlines the strength of the relationship between the MSTP and these important parameters of cardiovascular fitness: VO_2_peak (mL/kg/min), Ventilatory threshold 1 (VT1) and Ventilatory threshold 2 (VT2), which were all strongly and positively related to the MSTP score.

The Test of Likeability was applied after each defined test in the study to monitor the affective valence or emotional responses of the children completing the measures. The participants provided the highest number of ‘excellent’ responses for the BOT2 and the MSTP (*n* = 12, 48%), compared to 9 (37.5%) for the VO_2_peak and 10 (40%) for the 20-m MSRT. However, the Kruskal-Wallis test revealed that there were no significant differences in the responses provided in the Test of Likeability between the measures of BOT2, VO_2_peak, MSTP and 20-m MSRT (H = 5.718, DF = 3, *p* = 0.126).

## 4. Discussion

The salient findings of the present study were that a newly developed field-based assessment of cardiorespiratory fitness (i.e., the MSTP), that was sensitively designed for a pediatric population, was a very strong predictor of VO_2_peak and VT2 and a strong predictor of VT1: Three measures of cardiorespiratory performance obtained during criterion-graded exercise testing. This finding is important for clinical practice, given that the MSTP: (i) can be performed in an environment requiring less space (i.e., 10 m laps) than other field-based shuttle tests; (ii) requires no specialized or expensive equipment; (iii) offers group or individualized yet discrete assessment of cardiorespiratory fitness, given that the test is conducted over a defined period of time (three minutes) and the outcome measure (no. of beanbags in the trays) is not obviously visible to other children in the way that it is in “drop out” tests. The importance of these factors cannot be overstated, given the prevalence of childhood obesity with reduced cardiorespiratory fitness in children and the need to assess, monitor and potentially manage children’s exercise capacity using measures specifically designed for use with children.

VO_2_peak is a strong determinant of future all-cause mortality and also incidence of morbidity associated with increasingly prevalent lifestyle disorders (e.g., metabolic syndrome) [18] consequently, the clinical relevance of determining cardiorespiratory fitness is well established. There are many limitations to performing routine determination of VO_2_peak, including the burden of necessary expertise and specialized equipment, resulting in a reduced accessibility to criterion forms of assessment. The results of the present study suggest that the MSTP is a suitable alternative to the criterion-graded exercise test for determining cardiorespiratory fitness, which directly addresses our first study aim. Irrespective of adiposity, age, or gender, the number of beanbags transported over three minutes during the MSTP was significantly and positively related to the participants VO_2_peak determined during treadmill testing, suggesting that the three minutes of this maximal intensity exercise was enough to drive the levels of VO_2_ close to peak levels. Moreover, the relative strength, linearity, and narrow confidence intervals of the relationship between the MSTP score and VO_2_peak (see Figure 1) confirm that this method of assessing cardiorespiratory fitness does not present systematic bias and is suitable across the range of cardiorespiratory fitness capacities typical for pediatric and adolescent cohorts. These results were comparable to previous studies with children. For example, earlier studies developed VO_2_peak prediction equations from the 20-m MSRT that accounted for 68–88% of the variance of VO_2_max when different combinations of sex, age and anthropometric data were included [10,19,20,21,22]. 

A second aim of this study was to contrast the strength of the relationship between the MSTP and VO_2_peak with that of another commonly utilized field test: 20-m MSRT. In the present study, the MSTP as a raw measure was found to have a stronger relationship with VO_2_peak than the 20-m MSRT, even when applying Leger et al.’s predictive equation [10] to estimate VO_2_max from the 20-m MSRT by factoring in gender (see Table 3). While previous studies have demonstrated that other field-based assessment methods (e.g., 20-m MSRT/BEEP Test; 2.4 km run etc.) can be effective for estimating cardiorespiratory fitness, these assessments tend to rely upon outcome measures that may negatively impact upon well-being and self-worth among those participants who perform poorly. An obese child, for example, who is also a slow runner may “drop out” of the shuttle-based tests earlier than classmates or take a much longer time to complete a fixed distance assessment, which would ultimately and directly lead to peer recognition of their poor performance. The MSTP, on the other hand, enables an individual’s cardiorespiratory fitness to be assessed in a more discrete manner, yet provides an outcome measure that, based on the findings of this study, is more strongly associated with the criterion measured VO_2_peak when compared with existing field-based assessments. An additional benefit to using the MSTP over other field-based tests is its moderate-to-very strong predictive validity of VT1 and VT2. These measures are known to be important predictors of health but also performance-related fitness such as tactical and technical motor skill proficiency [23]. Furthermore, with our results from the Test of Likeability, showing that the children rated the motor skills tests (BOT2) and the MSTP (a task-oriented activity) as ‘excellent’ more than the 20-m MSRT and the VO_2_peak treadmill test, we suggest that the MSTP is not only a valid measure to use for predicting VO_2_peak, but it may be a more appropriate measure for use in general pediatric populations. Acute affective responses have been shown to predict future exercise adherence [24,25], so enhancing the affective valence during cardiorespiratory testing procedures with children may have positive longer-term outcomes related to future engagement in physical activity and exercise [25].

Limitations that have been reported in previous studies for investigating cardiorespiratory fitness measures include the sampling bias towards fit healthy children, who may be thought to enjoy the rigorous testing procedures of measuring cardiorespiratory fitness more than unfit children. However, the participants in this study were representative of the Australian population for both BMI and motor proficiency with approximately a quarter of participants overweight or obese and a mean overall BMI just above the 50th percentile [17]. The mean motor skill proficiency of the study group was slightly over the 50th percentile indicating average motor proficiency, suggesting that there was no sampling bias towards children with higher or lower mean motor scores (e.g., balance, coordination, or strength and agility) that may have contributed to higher or lower running economy. Further to defying the possibility of a sampling bias in our study, the mean VO_2_peak (43.8 ± 11.2 mL/kg/min) of our participants was compared to the means reported in previous studies using treadmill testing with children of similar age which ranged from 41–53 mL/kg/min [26,27]. These results support our assertion that there is no sampling bias towards fit, healthy children in our study. 

It is acknowledged by the authors that the MSTP involves some additional skills of grasping to pick up the bean bag and increased agility for turning and changing direction more often than the 20-m MSRT; however, these were purposeful inclusions in the protocol to better engage young children in the activity, as it creates a more task-oriented approach to the measure which was anticipated to promote engagement with the activity. Further to supporting the use of the MSTP is the consideration of the complexity of the reported equations for estimating VO_2_peak from the 20-m MSRT. Previous prediction equations have all used complex mathematical systems/formulas to determine VO_2_peak [10,11,12,13,14,15,16,17,18,19,20]. As in the present study, gender, age, body mass and height were not significant predictors of VO_2_peak in the presence of MSTP, these variables are not required in the MSTP equation for estimating VO_2_peak expressed relative to body mass. This means that the MSTP equation is simpler and can be easily and quickly calculated without having to undertake other measures such as height, body mass, BMI, or skin folds. This is noteworthy, as the process of taking body measurements in school environments is a sensitive issue and with studies reporting cardiorespiratory fitness as a health variable that is independently associated with clustered cardiovascular disease risks [28], the need to take body measurements in schools to assess cardiovascular health status could potentially be eliminated by using the MSTP.

## 5. Conclusions

Based on the findings of this cross-sectional pilot study, the MSTP was found to be a valid measure of cardiorespiratory fitness with a very high predictive validity for estimating VO_2_peak in children using a simple-to-apply equation. Data from this study suggest that the MSTP could be considered, as a more appropriate (valid and suitable) measure to use than the 20-m MSRT for predicting VO_2_peak in child and adolescent populations, particularly considering the sensitivities of measuring cardiorespiratory fitness in groups of children with diverse fitness abilities (e.g., school environments).

### Recommendations and Implications for Future Research

Further research is required to establish the test-retest reliability of the MSTP and its external validity, to determine the extent to which the results of this pilot study can be generalized to children outside of the study group and to other populations (i.e., adults). Additionally, a larger cross-sectional study is required to develop normative values for the MSTP and the findings of this pilot study can be used in planning such investigations. Overall, the MSTP appears to be a valid and alternative measure which could be considered for use when assessing cardiorespiratory fitness in children, particularly when working with sensitive populations in large groups.

## Figures and Tables

**Table 1:** Physiological and anthropometric characteristics of study participants and correlations with cardiorespiratory fitness measures.

Characteristics	Mean ± SD	Pearson Correlations with Cardiorespiratory Fitness Measures		
		VO_2_Peak (mL/kg/min) (*n* = 24)	MSTP (no.) (*n* = 25)	20-m MSRT (no.) (*n* = 25)
		r	r	r
Age (year)	12.58 ± 2.68	−0.111 (0.605)	0.042 (0.840)	0.394 (0.051)
Height (cm)	158.56 ± 0.24	−0.252 (0.234)	−0.098 (0.643)	0.282 (0.172)
Body Mass (kg)	56.15 ± 34.30	−0.662 ** (<0.001)	−0.653 ** (<0.001)	−0.233 (0.261)
BMI (kg/m^2^)	20.96 ± 8.75	−0.728 ** (<0.001)	−0.766 ** (<0.001)	−0.382 (0.059)
BMI (Percentile)	51.84 ± 33.94	−0.590 ** (0.002)	−0.590 ** (0.002)	−0.290 (0.159)

VO_2_Peak: Peak Oxygen Consumption, MSTP: Modified Shuttle Test-Paeds, 20-m MSRT: 20 m Multi Stage Run Test. r = Pearson’s Correlation Coefficient (significance level is shown in brackets). ** Pearson Correlation is significant at the 0.01 level (2-tailed).

**Table 2:** Cardiorespiratory fitness characteristics of study participants during incremental exercise testing and field tests.

Variable	Mean ± SD (*n* = 24)
VO_2_peak (mL/kg/min)	43.8 ± 11.2
VT1 (mL/kg/min)	26.2 ± 7.8
VT2 (mL/kg/min)	37.1 ± 9.2
HRpeak (beats/min)	190 ± 12
RERpeak	1.12 ± 0.11
MSTP Total Score (No.)	22.10 ± 3.05
20-m MSRT Level (No.)	5.48 ± 2.96
Predicted VO_2_max (mL/kg/min)	45.93 ± 7.71

Data presented as mean ± SD. VO_2_peak: peak oxygen uptake, VT: ventilatory threshold, HRpeak: peak exercise heart rate, RERpeak: peak respiratory exchange ratio, MSTP: Modified Shuttle Test-Paeds, 20-m MSRT: 20-m Multistage Running Test, Predicted VO_2_max from 20-m MSRT using Leger’s predictive equation [10].

**Table 3:** Pearson’s correlations (r) and regression coefficients (r^2^) between VO_2_peak (mL/kg/min) and alternative field tests for cardiorespiratory fitness.

Variable	VO_2_peak (mL/kg/min) For Study Participants (*n* = 24)	
	r	r^2^
MSTP (No.)	0.866 ** (<0.001)	0.749 ^β^ (<0.001)
20-m MSRT (No.)	0.697 ** (<0.001)	0.486 ^β^ (<0.001)
Predicted VO_2_max from 20-m MSRT equation	0.780 ** (<0.001)	0.608 ^β^ (<0.001)

For Pearson’s Correlations and Regression Coefficients, the level of significance for each value is shown in brackets. MSTP: Modified Shuttle Test-Paeds; 20-m MSRT: 20-m Multistage Running Test. Predicted VO_2_max from 20-m MSRT using the Leger et al.’s predictive equation [10]. ** Pearson Correlation is significant at the 0.01 level (2-tailed). ^β^ Regression Coefficient is significant at the 0.05 level.
